# High diagnostic yield of line probe assay as a point-of care test in a TB rural setting

**DOI:** 10.1186/1471-2334-14-S3-P40

**Published:** 2014-05-27

**Authors:** Aarti Kotwal, Rajiv Kumar Agarwal, Biswaroop Chatterjee, Barnali Kakati, Pronoti Sarkar, Sudhir Singh, Dolly Pokhriyal, Bhupendra Singh Chauhan

**Affiliations:** 1 Department of Microbiology, Himalayan Institute of Medical Sciences, Jolly Grant, Dehradun, India

## Background

Tuberculosis is the second leading cause of death due to infectious disease worldwide. Delay in bacilli isolation in culture, cumbersome susceptibility testing methods, lack of methods for differentiation of *Mycobacterium tuberculosis* (MTB) from non tuberculous mycobacteria, are important issues which impinge on the WHO’s millennium goal of disease reduction by 2015. Traditional methods of TB detection and anti tB drug susceptibility assays are time consuming and with a steep rise in number of MDR tB cases the need of the hour is rapid diagnostic methods like direct drug susceptibility testing in liquid medium, molecular hybridization methods and RealTime PCR.

## Methods

This study was conducted to assess the efficacy of line probe assay in detecting MTB from smear negative culture negative samples in a tertiary care centre catering to the need of a high burden TB population of Uttarakhand.

## Results

Between January 2013 and November 2013 a 22.7%positivity rate of sputum samples by fluorescent staining was reported. Culture by MGIT (Mycobacterium growth indicator tube) of 162 smear negative samples from patients clinically or radiologically pulmonary TB suspects was performed and 78(48.1%) samples grew MTB. Further, 50 samples which were smear negative and MGIT negative from patients with strong suspicion of pulmonary tuberculosis were tested by line probe assay and 34(68%) samples turned out to be positive.

## Conclusion

Newer methods offer definite improvements in the ability to detect MTB. However, there is a strong need to deploy these tests in areas where MTB burden is highest.

